# Increase in polymorphonuclear myeloid-derived suppressor cells and regulatory T-cells in children with B-cell acute lymphoblastic leukemia

**DOI:** 10.1038/s41598-021-94469-x

**Published:** 2021-07-22

**Authors:** Asmaa M. Zahran, Azza Shibl, Amal Rayan, Mohamed Alaa Eldeen Hassan Mohamed, Amira M. M. Osman, Khaled Saad, Khaled Hashim Mahmoud, Aliaa M. A. Ghandour, Khalid I. Elsayh, Omnia El-Badawy

**Affiliations:** 1 Department of Clinical Pathology, South Egypt Cancer Institute, Assiut University, Assiut, Egypt; 2 Pediatric Oncology Department, South Egypt Cancer Institute, Assiut University, Assiut, Egypt; 3 Clinical Oncology Department, Faculty of Medicine, Assiut University, Assiut, 71516 Egypt; 4 Pediatric Department, Faculty of Medicine, Assiut University, Assiut, Egypt; 5 Medical Microbiology and Immunology Department, Faculty of Medicine, Assiut University, Assiut, Egypt

**Keywords:** Paediatric cancer, Medical research, Oncology

## Abstract

Our study aimed to evaluate the levels of MDSCs and Tregs in pediatric B-cell acute lymphoblastic leukemia (B-ALL), their relation to patients’ clinical and laboratory features, and the impact of these cells on the induction response. This study included 31 pediatric B-ALL patients and 27 healthy controls. All patients were treated according to the protocols of the modified St. Jude Children’s Research Hospital total therapy study XIIIB for ALL. Levels of MDSCs and Tregs were analyzed using flow cytometry. We observed a reduction in the levels of CD4 + T-cells and an increase in both the polymorphonuclear MDSCs (PMN-MDSCs) and Tregs. The frequencies of PMN-MDSCs and Tregs were directly related to the levels of peripheral and bone marrow blast cells and CD34 + cells. Complete postinduction remission was associated with reduced percentages of PMN-MDSCs and Tregs, with the level of PMN-MDCs in this subpopulation approaching that of healthy controls. PMN-MDSCs and Tregs jointly play a critical role in maintaining an immune-suppressive state suitable for B-ALL tumor progression. Thereby, they could be independent predictors of B-ALL progress, and finely targeting both PMN-MDSCs and Tregs may be a promising approach for the treatment of B-ALL.

## Introduction

Acute lymphoblastic leukemia (ALL), a hematologic malignancy characterized by the malignant clonal proliferation of lymphoid progenitors^1^, is considered to be the most common childhood cancer. One of the well-known mechanisms by which tumor cells evade the immune system is the induction of antigen-specific unresponsiveness. It has become clear that tumor-specific T-cells are inhibited and become anergic in the tumor microenvironment^2–5^. Nevertheless, the mechanisms behind such tumor-induced immune suppression remain ill-defined^6^. Myeloid-derived suppressor cells (MDSCs) have become the focus of intense study in recent years, with much of our knowledge on the role of MDSCs in tumors driven by murine experiments^7^. MDSCs are a varied population of immature myeloid cells with strong suppressing functions of T- and natural killer cells^8,9^ and are able to overwhelm T-cell proliferation and immunological functions in patients with different types of tumors^10–14^. MDSCs consist of the following two main subpopulations stratified according to their phenotypical and morphological characteristics: monocytic MDSCs (MO-MDSCs) and polymorphonuclear MDSCs (PMN-MDSCs)^15^, with the latter previously known as granulocytic MDSCs (G-MDSCs)^16^. Both MDSC subsets have been identified in different pathological conditions in the bone marrow, peripheral blood, and tumor tissue, where PMN-MDSCs represent more than 80% of all MDSCs in most cancer entities^17^. MDSCs have interwoven relations with other immune cells, leading the host immune system to adopt an immune-suppressive and tolerogenic status^18^. Regulatory T-cells (Tregs) represent a subset of T lymphocytes that play a crucial role in maintaining tolerance^19^, thus resembling MDSCs. Tregs’ central mechanism of tumor evasion can lead to human cancer progression and may contribute to the failure of immunotherapy in cancer patients^20,21^. Several studies have reported an increase in percentages of Tregs in the peripheral blood of patients with solid tumors and hematologic malignancies relative to in healthy controls^22–24^.

Accumulating evidence suggests that the profound immunosuppression observed in patients with leukemias is attributable to increased levels and activity of MDSCs and Tregs^25–30^. However, their role in childhood B-cell acute lymphoblastic leukemia (B-ALL) has not been comprehensively studied except for in a few preliminary reports^31–33^. Moreover, their status and function in ALL are yet to be elucidated^33,34^. It has not been established whether a possible communication between MDSCs and Tregs exists, causing tumor progression^6^.

The reciprocal relation of MDSCs and Tregs is a topic of intense exploration, as these cells have a coordinated capability of suppressing host immunity^18^. However, scarce studies have investigated this relation in leukemias, especially of the ALL type^33,35–37^. Clarification of these relations may offer better insights for tailoring immunotherapy for those patients.

## AIMS

The present work aimed to evaluate the levels of MDSCs and Tregs in pediatric B-ALL, their relation to patients’ clinical and laboratory features, and the impact of these cells on the induction response.

## Methods

Thirty-one newly diagnosed pediatric precursor B-ALL patients (≤ 18 years) and 27 age- and sex-matched healthy controls were recruited from the Pediatric Oncology Department of South Egypt Cancer Institute, Assiut University. The Ethical Committee of Assiut University approved all procedures in our research. All protocols and investigations of our study followed the regulations of the research ethics committee of Assiut University (No. 1-2018). Informed written consent was obtained from all guardians of children included in the study.

Children who received steroids or chemotherapy before enrollment in this study were excluded. The study was reviewed and approved by the institutional review board and informed consent for study inclusion was obtained from the patients’ parents or guardians.

### Diagnosis of B-ALL

The diagnosis of ALL among patients included in this study relied primarily on the morphologic and cytochemical features of bone marrow smears and immunophenotyping of leukemic blast cells (based on the World Health Organization classification)^38^. Patients with precursor B-ALL were recruited into this study. Central nervous system (CNS) involvement was confirmed through a lumbar puncture and cerebrospinal fluid (CSF) cytological examination performed on the first day of induction treatment, separate from the timing of diagnostic bone marrow aspiration so as to avoid traumatic lumbar puncture because thrombocytopenia resulting from traumatic lumbar puncture may trigger a serious risk of later CNS relapse. Following sample collection, the CNS status of each patient was determined, according to Smith's classification; specifically, CNS leukemia was defined as the presence of leukemic blasts and white blood cell count of at least 5/µL^39^. The presence of bulky disease was detected by computed tomography (CT) and referred to the existence of a mediastinal mass measuring at least one-third in the intrathoracic dimension, solitary lymph node measuring at least 3 cm or splenomegaly below the umbilicus^40^.

### Risk stratification and treatment

According to the National Cancer Institute/Rome criteria ^39,41^, patients were classified into standard- and high-risk groups, where the former were those aged one to nine years with an initial total leukocyte count (TLC) of less than 50 × 10^9^/L; had a DNA index of 1.16 or more; without CNS-3 status, testicular leukemia (documented by ultrasonographic examination), t(9;22), t(4;11), t(1;19) associated with a pre-B immunophenotype, an *MLL* gene rearrangement, or near-haploidy; and bone marrow should not contain 5% or more leukemic blasts on day 15 of remission induction, while the remaining were considered high-risk patients, including those with leukemia with CNS involvement confirmed at diagnosis. All patients were treated according to the modified St. Jude Children’s Research Hospital total therapy study XIIIB protocol for ALL^41^, which involves an induction phase with a six-drug regimen (vincristine, dexamethasone, asparaginase, daunorubicin, Ara-C, and etoposide) for 29 days after a prephase of four days of steroids, followed by bone marrow aspiration and CSF cytology to evaluate the postinduction response. All patients received consolidation therapy with 2 weekly doses of high-dose methotrexate followed by leucovorin rescue beginning 42 h later and mercaptopurine for 2 weeks. Postremission therapy (120 weeks) for lower-risk cases consisted of daily mercaptopurine and weekly intravenous methotrexate, reinforced by high-dose methotrexate and mercaptopurine (similar doses as used in consolidation therapy) every 8 weeks in the first year, and dexamethasone and vincristine pulses every 4 weeks, in addition re-induction therapy (similar to the initial 4-week remission induction with only one dose of etoposide plus cytarabine on day 22, followed by 2 weekly doses of high-dose methotrexate plus daily mercaptopurine) was administered from weeks 16 to 21 after remission. Altogether, 10 courses of high-dose methotrexate were given. All higher-risk cases were then transferred to a continuation phase in which drug pairs were administered in a weekly rotation arrangement for 120 weeks with re-induction treatment was the same as that used in lower-risk cases, also a total of 10 courses of high-dose methotrexate were given. Bone marrow aspiration and CSF cytology were performed during the continuation phase every two to three months to detect any relapse. Complete remission was confirmed by the finding of less than 5% blasts in an adequately cellular marrow (≥ 20%) on bone marrow aspiration together with hematologic recovery (absence of peripheral blood blasts, and recovery of neutrophil and platelet counts above 1 *10^9^ /l and 100* 10^9^ /l, respectively), and the absence of extramedullary disease. Conversely, Induction failure was defined as failure to achieve morphologic complete remission with < 5% bone marrow blasts^42^.

### Flow cytometric detection of regulatory T-cells

**Figure 1:**
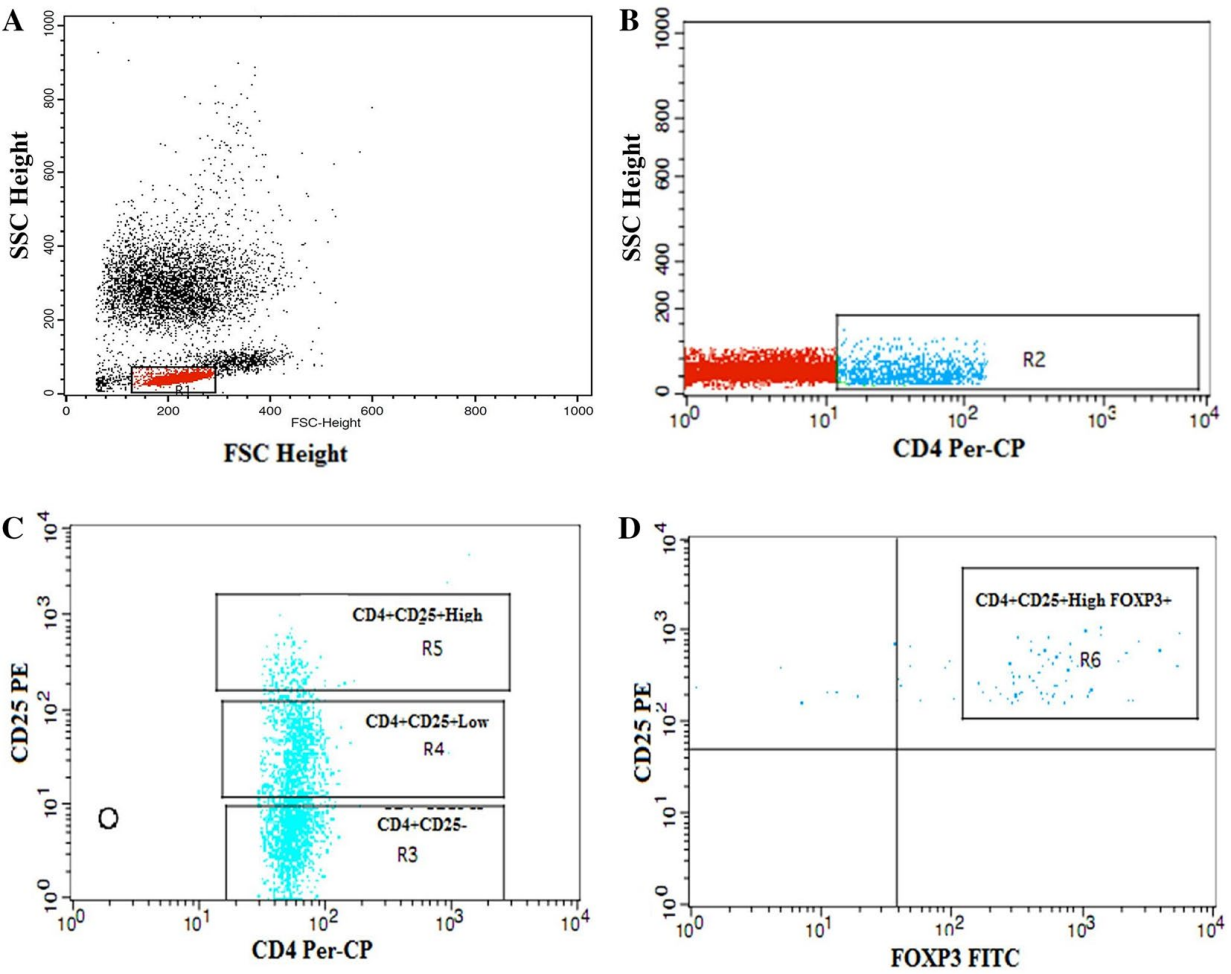
Flow cytometric detection of regulatory T cells. (**A**) The lymphocyte population was defined on forward, and side scatter histogram (R1). (**B**) The expression of CD4 on the lymphocytes population was detected, then CD4^+^ cells were gated for further analysis of CD25. (**C**) Three gates were drown to define CD4^+^CD25^-^ cells, CD4^+^CD25^+low^ cells and CD4^+^CD25^+high^ cells. (**D**) The percentage of CD4^+^CD25^+high^Foxp3^+^cells (regulatory T cells) was then assessed.

Regulatory T-cells were enumerated using fluoroisothiocyanate-conjugated Foxp3 (IQ Products, Groningen, the Netherlands), phycoerythrin-conjugated cluster of differentiation (CD)25, and peridinium–chlorophyll–protein complex (Per-CP)–conjugated CD4 (BD Biosciences, San Jose, CA, USA). The methodology of Tregs detection have been previously described in detail^43^. Total CD4 + CD25 + , CD4 + CD25 + low, CD4 + CD25 + high and CD4 + CD25 + high Foxp3 + Tregs was evaluated as percentages of CD4 + cells as shown in Fig. 1.

### Flow cytometric detection of myeloid-derived suppressor cells

**Figure 2:**
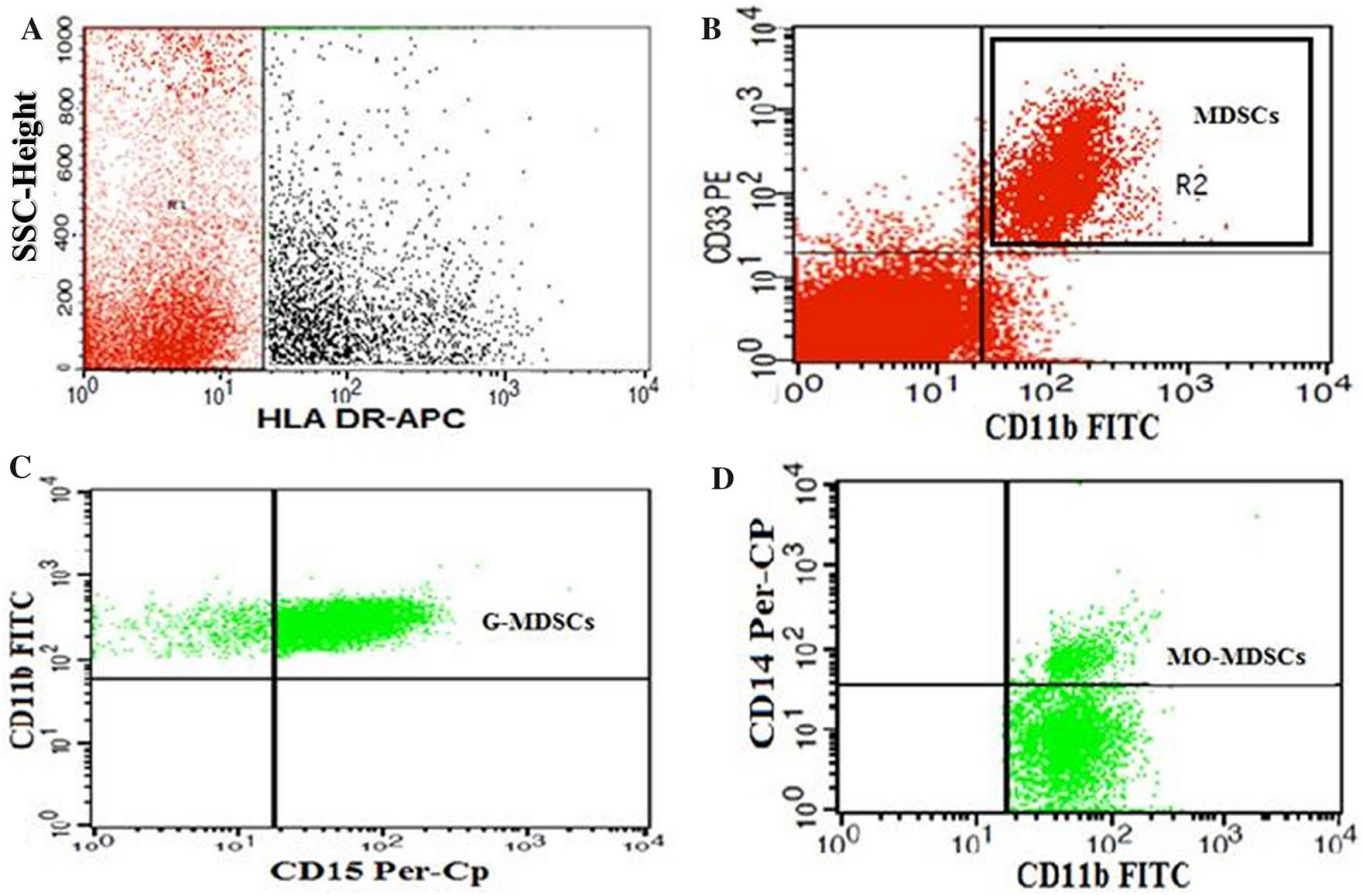
Flow cytometric detection of myeloid-derived suppressor cells. (**A**) HLA-DR negative cells (R1) were selected from HLA-DR and side scatter histogram. (**B**) HLA-DR negative cells were assessed for their expression of CD33 and CD11b to detect total myeloid-derived suppressor cells (MDSCs: HLA-DR^-^CD33^+^CD11b^+^). (**C,D**) Total myeloid-derived suppressor cells were assessed for their expression of CD15 and CD14 to detect monocytic myeloid-derived suppressor cells (MO-MDSCs: HLA-DR-CD33^+^CD11b^+^CD14^+^), and polymorphonuclear myeloid-derived suppressor cells (PMN-MDSCs “previously G-MDSCs”: HLA-DR^-^CD33^+^CD11b^+^ CD15^+^).

MDSCs were detected by using fluoroisothiocyanate-conjugated CD11b, phycoerythrin-conjugated CD33, Per-CP–conjugated CD15, Per-CP–conjugated CD14, and allophycocyanin-conjugated HLA-DR (all purchased from BD Biosciences), as shown in Fig. 2. The methodology of MDSCs detection have been previously described in detail^44^. HLA-DR–negative cells were assessed for their expression of both CD33 and CD11b to detect total MDSCs (MDSCs: HLA-DR − CD33 + CD11b +), then were further evaluated for their expression levels of CD15 and CD14 to identify MO-MDSCs (HLA-DR − CD33 + CD11b + CD14 +) and PMN-MDSCs (HLA-DR − CD33 + CD11b + CD15 +). M-MDSCs and PMN-MDSCs were expressed as percentages of total MDSCs.

### Statistical analysis

The Statistical Package for the Social Sciences, version 16.0 (IBM Corporation, Armonk, NY, USA) was used for all statistical analyses. Results were presented as mean ± standard deviation (SD) or standard error (SE) values. The Student's t-test was used to determine statistically significant differences in tested cells between the patient and control groups. The Mann–Whitney U test and one-way analysis of variance were used to compare between patient subgroups. Associations between the variables were explored using Pearson’s correlation. A p-value of less than 0.05 was considered to be statistically significant.

## Results

### Patients’ characteristics

This study included 31 children with B-ALL with a mean age of 7 ± 0.7 years, who were further divided into three age groups for investigation. Patients’ demographic and clinical characteristics are summarized in Table 1. A mediastinal mass was detectable in seven (23%) patients, while six (19%) showed CNS infiltration and 11 (35%) presented with extramedullary bulky disease. Of the 31 study participants, 17 (55%) had high-risk ALL.

**Table 1:** Main characteristics of B-cell acute lymphoblastic leukemia patients.

Parameters	Patients
Age (y)*	7 ± 0.7
< 1	2 (6.5%)
1– < 10	17 (54.8%)
≥ 10	12 (38.7%)
Sex Male	18 (58%)
Female	13 (42%)
PB blast cells (%)*	21 ± 2
BM blast cells (%)*	52.2 ± 4
CD34^+^ cells (%)*	46.4 ± 4
CD19^+^ in BM (%)*	57 ± 20
HB (g/dL)*	7.2 ± 0.3
TLC (10^9^/L)*	31 ± 9
< 50	18 (58%)
≥ 50	13 (42%)
lymphocytes (%)	34.9 ± 9
Platelets (× 10^9^/L)	40.4 ± 4
Mediastinal mass	7 (23%)
CNS infiltration	6 (19%)
Bulky disease	11 (35%)
**NCI/Rome risk**	
Standard	14 (45%)
High	17 (55%)

*PB* peripheral blood, *BM* bone marrow, *HB* hemoglobin, *TLC* total leukocyte count, *CNS* cerebral nervous system, *NCI* National Cancer Institute Standard risk: age 1– < 10 with initial TLC < 50 × 10^9^/L, High risk: all other patients, including patients with CNS leukemia at diagnosis. Results expressed as number (percentage), * expressed as mean ± SD.

### Levels of peripheral Tregs and MDSCs among B-ALL patients and controls

**Figure 3:**
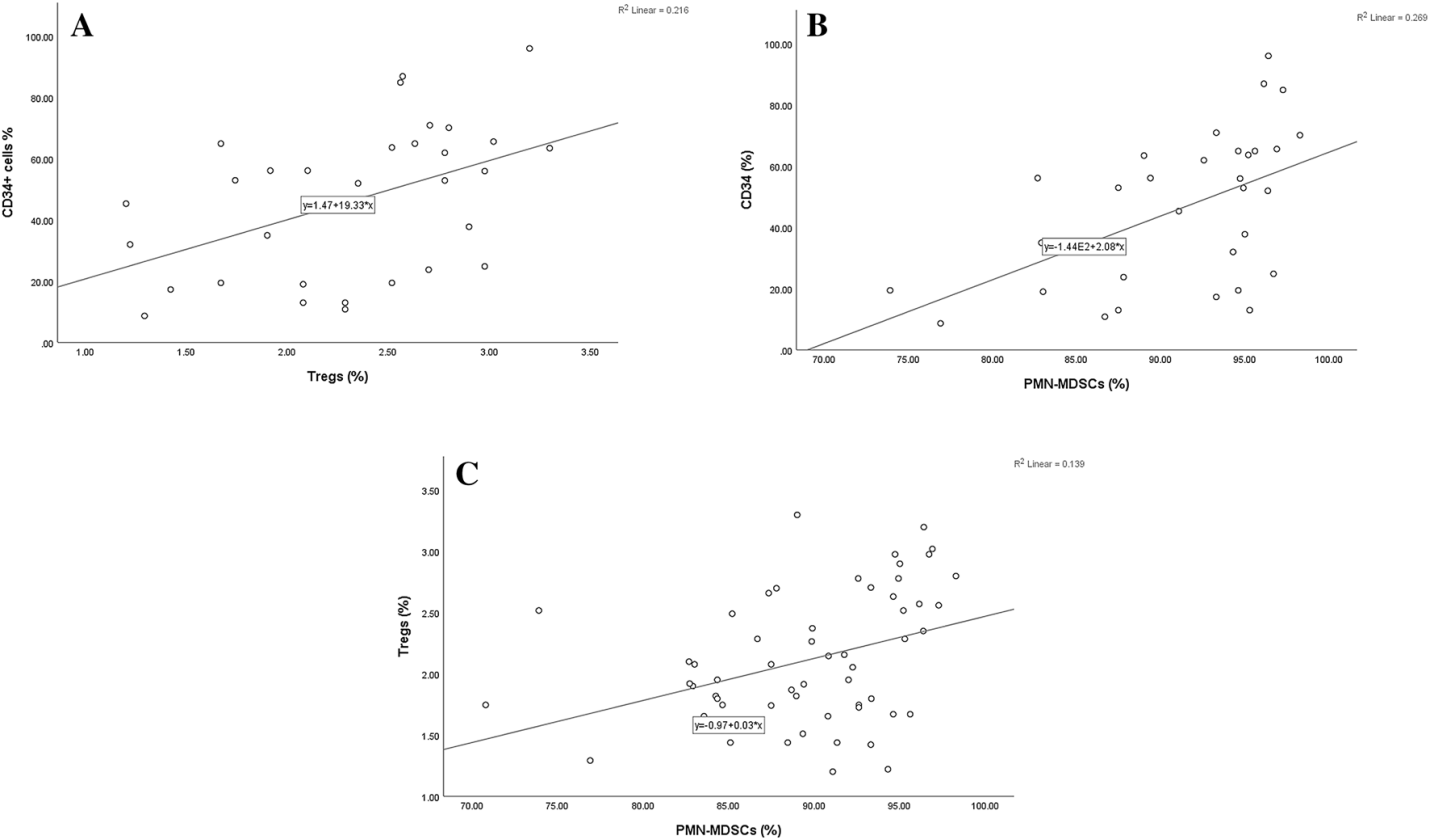
CD34^+^ cells showing direct relations with the levels of Tregs in (**A**) and MDSCs in (**B**) and a direct association between the levels of Tregs and PMN-MDSCs was shown in (**C**).

As shown in Table 2, a significant elevation of total MDSCs was observed in the B-ALL patients relative to among the healthy controls. However, this increase was found only in those with the polymorphonuclear subtype of MDSCs. While a noticeable reduction was detected in the levels of CD4 + T-cells, including those with low expression of CD25, a highly significant increase was observed in the levels of CD4 + T-cells with high expression of CD25 and Tregs in B-ALL patients in comparison within the control group. Moreover, the levels of Tregs were directly related to those of PMN-MDSCs (r = 0.3; p = 0.03) (Fig. 3).

**Table 2:** Higher frequencies of peripheral polymorphonuclear myeloid-derived suppressor cells and regulatory T cells are detected in children with B-acute lymphoblastic leukemia.

Cells	Patients (n = 31)	Control (n = 27)	*p-*value
Total MDSCs (%)	4 ± 0.4	1.3 ± 0.1	** < 0.0001**
PMN-MDSCs (%)	91.3 ± 1	87.5 ± 1	**0.01**
MO-MDSCs (%)	9.4 ± 0.8	11.2 ± 0.8	0.1
CD4^+^(%)	28.9 ± 1	40.6 ± 1	** < 0.0001**
CD4^+^CD25^+low^ (%)	17.8 ± 0.5	21.4 ± 0.5	** < 0.0001**
CD4^+^CD25^+high^ (%)	7.2 ± 0.5	4.9 ± 0.2	** < 0.0001**
CD4^+^CD25^+high^FoxP3^+^ (Tregs)(%)	2.3 ± 0.1	1.9 ± 0.1	** < 0.0001**

*n* number, *MDSCs* myeloid-derived suppressor cells, *PMN-MDSCs* Polymorphonuclear myeloid-derived suppressor cells, *MO-MDSCs* monocytic myeloid-derived suppressor cells, *Tregs* regulatory T cells. Results expressed as mean ± SE. Student's t-test; Significant *p-*value < 0.05.

### Relationship between the levels of peripheral Tregs and MDSCs with initial blast characteristics

As presented in Table 3 and Fig. 3, both the frequencies of Tregs and PMN-MDSCs were directly influenced by the percentages of PB and BM blast cells and CD34 + cells. Moreover, the level of CD34 + cells showed a weak positive correlation with that of CD4 + CD25low T-cells and negative associations with those of MO-MDSCs and CD4 + T-cells, respectively.

**Table 3:** Relations of peripheral myeloid-derived suppressor cells and regulatory T cells with blast cells in children with B-cell acute lymphoblastic leukemia.

Cells	PB blast cells	BM blast cells	CD34^+^ cells
Total MDSC	r = 0.1	r = 0.1	r = 0.07
	*p* = 0.3	*p* = 0.2	*p* = 0.4
PMN–MDSCs	**r = 0.4**	**r = 0.4**	**r = 0.5**
	***p* = 0.007**	***p* = 0.006**	***p* = 0.001**
MO-MDSCs	r = − 0.2	r = − 0.2	r = − 0.2
	*p* = 0.1	*p* = 0.1	*p* = 0.06
CD4^+^T cells	r = − 0.2	r = − 0.1	**r = **− **0.3**
	*p* = 0.2	*p* = 0.2	***p* = 0.03**
CD4^+^CD25^+low^	r = 0.2	r = 0.3	**r = 0.3**
	*p* = 0.1	*p* = 0.08	***p* = 0.03**
CD4^+^CD25^+high^	r = − 0.2	r = − 0.07	r = − 0.2
	*p* = 0.2	*p* = 0.4	*p* = 0.2
CD4^+^CD25^+high^FoxP3^+^ (Tregs)	**r = 0.5**	**r = 0.6**	**r = 0.5**
	***p* = 0.003**	***p* = 0.001**	***p* = 0.004**

*PB* peripheral blood, *BM* bone marrow, *MDSCs* myeloid-derived suppressor cells, *PMN—MDSCs*: polymorphonuclear myeloid-derived suppressor cells, *MO-MDSCs* monocytic myeloid-derived suppressor cells, *Tregs* regulatory T cells. Pearson correlation, r Pearson’s correlation coefficient,

significant *p-*value < 0.05.

### Relationship between the levels of peripheral Tregs and MDSCs and prognostic features

The levels of Tregs and MDSCs at diagnosis in correlation with patients’ prognostic factors, including age, sex, TLC, mediastinal mass, CNS involvement at diagnosis, and bulky disease, were examined. Ultimately, no significant differences were observed in the levels of CD4 + T-cells, Tregs, total MDSCs, and MDSC subtypes between patients with standard-risk and high-risk B-ALL. Similarly, no differences were found in the percentages of those cells between male and female patients and among the three different age groups. However, a weak correlation was observed between age and the percentage of PMN-MDSCs (r = 0.38; p = 0.02). Moreover, the frequency of CD4 + CD25 + high T-cells was significantly higher in B-ALL children with a TLC of at least 50 × 10^9^/L as compared with among those with a TLC of less than 50 × 10^9^/L. Also, a correlation was detected between the frequency of CD4 + CD25 + high T-cells and the TLC (r = 0.4; p = 0.007). Otherwise, no major differences were observed between the above groups of patients concerning the levels of other tested cell subsets.

### Relationship between the levels of peripheral Tregs and MDSCs and the postinduction response

**Figure 4:**
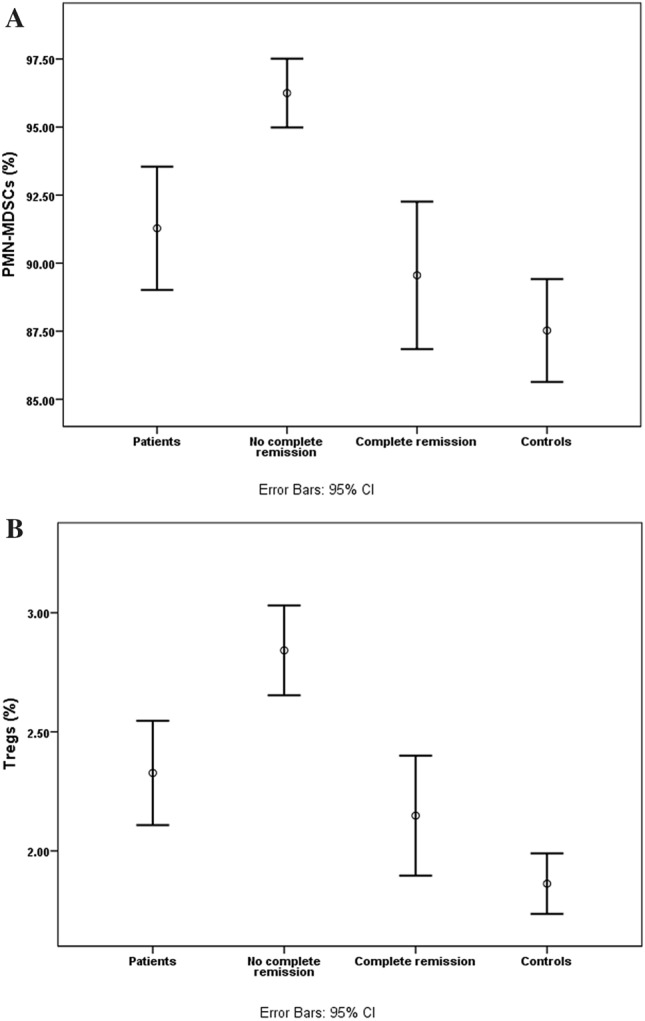
Different levels of (**A**) PMN-MDSCs and (**B**) Tregs among B-ALL patients, patients who did not undergo complete remission, patients showing complete post-induction remission, and healthy controls.

As shown in Table 4 and Fig. 4, a significant drop in the percentage of PMN-MDSCs in patients showing complete postinduction remission was observed relative to those who did not experience complete remission, even approaching the level in the healthy controls (p = 0.2). Likewise, Treg levels were decreased considerably in patients showing complete postinduction remission as compared to those who did not undergo complete remission, although they remained significantly higher than their levels in healthy controls (p = 0.03).

**Table 4:** Relation of myeloid-derived suppressor cells and regulatory T cells with the post-induction response in children with B-cell acute lymphoblastic leukemia.

Cells	Complete remission (n = 23)	No complete remission (n = 8)	*p-*value
Total MDSC (%)	3.8 ± 0.4	4.5 ± 1	0.6
PMN-MDSCs (%)	89.6 ± 1	96.2 ± 0.5	** < 0.0001**
MO-MDSCs (%)	10.1 ± 0.9	7.2 ± 1	0.06
CD4^+^ (%)	29.6 ± 1	27 ± 3	0.3
CD4^+^CD25^+low^ (%)	17.6 ± 0.6	18.3 ± 0.7	0.4
CD4^+^CD25^+high^ (%)	7 ± 0.6	6.7 ± 1	0.3
CD4^+^CD25^+high^FoxP3^+^(Tregs) (%)	2.1 ± 0.1	2.8 ± 0.1	**0.001**

*n* number, *MDSCs* myeloid-derived suppressor cells, *PMN-MDSCs* polymorphonuclearmyeloid-derived suppressor cells, *MO-MDSCs* monocytic myeloid-derived suppressor cells, *Tregs* regulatory T cells. Results expressed as mean ± S.E. Mann–Whitney U test; significant *p-*value < 0.05.

## Discussion

The etiology and immune pathogenesis of ALL remains unclear^45^. Immune suppression is a hallmark of most tumors and is essential for cancer growth and progression^46,47^. Although accumulating evidence suggests that Tregs and MDSCs are associated with immune suppression in many tumors, it has not been proven whether a possible relationship exists between MDSCs and Tregs during tumor progression^6^. This association may be because MDSC secretes immunosuppressive cytokines such as transforming growth factor–β and interleukin-10, which reduce the antitumor activity of effector T-cells and recruit Tregs^48,49^.

Studies on the roles of Tregs^31,32^, and MDSCs^33^ in B-ALL are limited and few to date have investigated the association between MDSCs and Tregs in leukemias^35–37^, especially of the ALL type^37^. Clarification of this relationship may offer better insights for tailoring immunotherapy for those patients. Thus, the present work aimed to evaluate the levels of MDSCs and Tregs in pediatric B-ALL, their relationship with patients’ clinical features, and the impact of these cells on the induction response.

In our study, we noticed a significant reduction in CD4 + T-cell levels and an increase in both the levels of PMN-MDSCs and Tregs. The frequencies of PMN-MDSCs and Tregs were directly correlated with the levels of PB and BM blast cells and CD34 + cells. Moreover, complete postinduction remission was associated with reduced percentages of the two former suppressor cells, with the level of PMN-MDCS even approaching that of the healthy controls in some cases. Nevertheless, neither MDSCs nor Tregs displayed a significant relationship with TLC and did not exhibit differences between standard and high-risk B-ALL patients. Tregs did show a direct association with PMN-MDSCs.

In agreement with our results, prior studies have reported significantly decreased CD4 + CD25 + cell populations amongst the PBMCs of B-ALL patients as compared with in healthy controls^50^. This immune suppression was endorsed by the accompanying elevation in the frequency Tregs and immunosuppressive potential with the advancement of malignancy in B-ALL patients. Additionally, a reduction in the level of Tregs was observed after chemotherapy, indicating the existence of an enhanced immune status^50^.

In the same way, Liu et al.^33^ reported significantly elevated levels of PMN-MDSCs in both the peripheral blood and bone marrow of patients with B-ALL as compared with in healthy controls. Also, these authors observed a marked decrease in the levels of PMN‐MDSCs among patients with B‐ALL entering remission after therapy, approaching the levels recorded in healthy controls, and noticed that these levels correlated with B‐ALL prognostic markers. On the contrary, levels of PMN-MDSCs in patients without remission remained higher after therapy relative to in healthy controls^33^. Likewise, earlier studies have reported that efficient immunotherapy was associated with a decline in MDSC frequency and activity^51,52^.

A direct association between the levels of Tregs and MDSCs has been reported in different types of leukemias^35–37^. Consistent with our findings, Salem et al.^37^ reported increased levels of both MDSCs and Tregs in pediatric B-ALL patients as compared with in healthy controls. They also reported that Tregs' levels gradually decreased after induction of chemotherapy but did not reach normal levels. Conversely, these authors noticed that the levels of MDSCs continued increasing markedly following the initiation of chemotherapy.

Limitations of the study: The need for proper evaluation of minimal residual disease is important for assessment of disease remission. Unfortunately, for financial causes, minimal residual disease was not part of the routine workup of the patients at the time of the study.

## Conclusions

Our findings agree with those of other studies suggesting that both PMN‐MDSCs and Tregs jointly play a critical role in maintaining an immune-suppressive state suitable for B‐ALL tumor progression. Thereby, they could be an independent predictor of B-ALL progress, and finely targeting both PMN‐MDSCs and Tregs may be a promising approach for the treatment of B‐ALL patients.
